# Performance of the Amsterdam IADL scale in Assessing Functional Decline among Older People in Primary Health Care

**DOI:** 10.1002/alz.093897

**Published:** 2025-01-09

**Authors:** Ayesha Fawad, Maria H Nilsson, Sietske A.M. Sikkes, Merel C. Postema, Erik Stomrud, Pontus Tideman, Oskar Hansson, Sebastian Palmqvist

**Affiliations:** ^1^ Clinical Memory Research Unit, Department of Clinical Sciences Malmö, Faculty of Medicine, Lund University, Lund, Sweden, Malmö Sweden; ^2^ Alzheimer Center/dpt Neurology, VU University Medical Center of Amsterdam, Amsterdam Neuroscience, De Boelelaan 1118., Amsterdam Netherlands; ^3^ Alzheimer Center Amsterdam, Neurology, Vrije Universiteit Amsterdam, Amsterdam UMC location VUmc, Amsterdam Netherlands; ^4^ Clinical Memory Research Unit, Department of Clinical Sciences Malmö, Faculty of Medicine, Lund University, Lund Sweden; ^5^ Clinical Memory Research Unit, Department of Clinical Sciences, Lund University, and Memory Clinic, Skåne University Hospital, Malmö Sweden

## Abstract

**Background:**

The Amsterdam Instrumental ADL Questionnaire (A‐IADL‐Q) has shown promising performance in detecting subtle impairment in IADL independency. Importantly, it has not been validated in older people in a primary care setting, i.e., where the initial work‐up and triaging of cognitive impairment occurs. The main aim was to examine the accuracy of the A‐IADL‐Q for estimating the cognitive stage in a primary care sample, both in the whole population and within the Alzheimer’s disease (AD) continuum. Secondary aims were to examine associations with the clinical dementia rating (CDR) scale and global cognition (MMSE), including the effects of non‐cognitive co‐morbidities and medications.

**Method:**

This cross‐sectional study includes informants of 362 participants from the BioFINDER‐Primary care study. They completed the A‐IADL‐Q (higher scores = “better”) either on an iPad or at home on a computer. Cognitive stage was assessed by a dementia expert (blinded to A‐IADL‐Q scores). Amyloid status was determined using FDA‐approved CSF or PET measures. The main outcome was cognitive stage according to the NIA‐AA scale. Secondary outcomes were CDR‐sum of the boxes (CDR‐SOB) and MMSE. Receiver Operating Curves (ROC) analyses were used to calculate the area under the curves (AUCs).

**Result:**

The mean age of participants (44.9% female) was 76.1 (SD 7.2) years. Out of which, 83 (23%) had subjective cognitive decline (SCD), 169 (47%) mild cognitive impairment (MCI), and 110 (30%) dementia. The AUC (95% CI) for differentiating between SCD versus MCI/dementia was 0.89 (0.85‐0.92), and it was 0.89 (0.85‐0.93) for SCD/MCI versus dementia. In amyloid positive participants (n = 303), AUCs were similar. Better A‐IADL‐Q scores were associated with worse results on CDR‐SOB (r = ‐0.73, p<0.001) and better global cognition (MMSE, r = 0.361, p<0.001). The effects of different co‐morbidities on the A‐IADL score, adjusted for cognitive stage, will be presented at AAIC.

**Conclusion:**

Our findings suggest that the A‐IADL‐Q accurately estimates the cognitive stage among cognitively impaired older people (with high prevalence of comorbidities) in a primary care setting. This can aid primary care physicians in the diagnosis of cognitive impairment, clinical management/support, and as part of the work‐up for identifying eligible participants for anti‐amyloid treatment.

